# Paediatric Society and Hyperinsulinism Charity National Surveys on CGM Access for Patients With Recurrent Hypoglycaemia

**DOI:** 10.1210/jendso/bvad021

**Published:** 2023-01-30

**Authors:** Sze May Ng, Sarah Dearman, Mark Fisher, Talat Mushtaq, Tabitha Randell

**Affiliations:** Paediatric Department, Southport and Ormskirk NHS Trust, Ormskirk L39 2AZ, UK; Department of Women's and Children's Health, University of Liverpool, Liverpool L69 3BX, UK; UK Children's Hyperinsulinism Charity, Registration no 1165562, London, UK; UK Children's Hyperinsulinism Charity, Registration no 1165562, London, UK; Department of Paediatric Endocrinology, Leeds Children's Hospital, Leeds LS1 3EX, UK; Department of Paediatric Endocrinology, Nottingham Children's Hospital, Nottingham NG15 1PB, UK

**Keywords:** continuous glucose monitoring, CGM, pediatric, hypoglycemia, hyperinsulinism

## Abstract

**Context:**

Recurrent hypoglycemia can result in significant neurological impairments in children and continuous glucose monitoring (CGM) technology has been shown to reduce recurrent hypoglycemia in conditions such as type 1 diabetes. In the United Kingdom, CGM devices are currently only recommended by the National Institute of Clinical Excellence (NICE) for patients with diabetes and not for other diagnoses.

**Objective:**

To examine access to CGM technology for children and young people with recurrent hypoglycemia in the United Kingdom.

**Methods:**

In 2021, the British Society of Paediatric Endocrinology and Diabetes (BSPED) conducted a national health professional survey in England, Wales, Scotland, and Northern Ireland looking at CGM access to funding for children and young people with recurrent hypoglycemia, without the diagnosis of diabetes. The UK Children's Hyperinsulinism Charity (UK CHC) also conducted a national patient survey.

**Results:**

Responses from BSPED were received from 55 units while the UK CHC received 69 responses from individual families, the largest response to a survey carried out by the charity. The results of the BSPED and UK CHC surveys found that funding streams for CGM were highly variable. Only 29% were able to access CGM for recurrent hypoglycemia and from these, 65% were self-funding CGM. Quality of life benefits were evident from the UK CHC survey on the utility of CGM in reducing worry, improving sleep, lessening the burden of frequently finger-pricking and reducing out-of-hours appointments as a result of hypoglycemia. Patient-reported utilization rates of blood glucose test strips per week were significantly reduced.

**Conclusion:**

BSPED and UK CHC national surveys support a call and a consideration for CGM access to be widened to patients who suffer from recurrent hypoglycemia such as those with hyperinsulinism or metabolic conditions. The prevention of recurrent hypoglycemia and improving quality of life for patients and carers remain a cornerstone management for people who suffer from frequent hypoglycemia. CGM education is critical to support its use and understand its limitations. Further research is warranted to determine the safety and efficacy of CGM in detection and reduction of hypoglycemic events, impact of hospital stay, and long-term neurological outcomes in those who suffer from recurrent hypoglceamia.

Continuous glucose monitoring (CGM) allows for continuous real-time blood glucose monitoring which reduces the need for frequent finger-prick blood testing [1]. CGM technology enables users to be warned of high or low blood glucose readings and provides trend data of blood glucose throughout the day. Current review suggests that CGM technology may offer the benefits in the detection and prevention of hypoglycemic episodes in conditions such as congenital hyperinsulinism, however, current limitations include high cost, delays in detection of hypoglyceamia and inaccuracies [1]. Hypoglycemia secondary to these conditions is serious, with almost 50% of children demonstrating neurological impairments as a result of recurrent hypoglycemic events [2]. The goal for optimal treatment management is to reduce hypoglycemia to near zero, to reduce overall exposure to symptomatic and asymptomatic hypoglycemia, to improve hypoglycemia awareness, and to reduce the fear of hypoglycemia in this population. The current standard of care for these patients are regular observations and intermittent fingerpick testing. However, this involves significant time burden on caregivers, and finger-prick tests provide no details of blood glucose trends with no alarm settings, and carers risk missing hypoglycemic events in-between infrequent tests.

CGM devices are only recommended by the UK National Institute of Clinical Excellence (NICE NG18 and NG17) for all patients with type 1 diabetes, and some people with type 2 diabetes who are on insulin, due to published literature on the achievement of targeted glycated hemoglobin (HbA1C) rather than reductions of hypoglycemic episodes as a clinical end point [3]. In the United Kingdom, there are now funding pathways in the National Health Service (NHS) for patients with diabetes on how CGM should be prescribed. However, CGM access has been reported to be highly variable and largely determined locally by Clinical Commissioning Groups (CCGs) or Integrated Care Boards (ICBs), and many cases still require individual funding applications (IFAs) to access CGM devices [4]. The aim of the national surveys is to advocate for a change in current policy to allow equitable access of CGM to patients suffering with recurrent hypoglycemia.

## Methods

From 1 January 2021 to 30 February 2021, the UK British Society of Paediatric Endocrinology and Diabetes (BSPED) conducted a national survey in England, Wales, Scotland, and Northern Ireland. These included all of the 22 specialist paediatric endocrine centres in England, Scotland, Wales, and Northern Ireland registered under the BSPED national endocrine peer review programme and a further 33 district general hospitals that manage patients with recurrent hypoglycemia.

### Ethics and Data Collection

The UK Children's Hyperinsulinism Charity (UK CHC) also conducted a survey in England, Wales, Scotland, and Northern Ireland between 12 October 2021 and 25 November 2021. The survey was posted within the UK CHC families support group via their private Facebook group platform. All families who participated in the survey consented and were made aware of the purpose of the questionnaire, with whom the data would be shared, and the criteria of eligibility to complete the survey prior to completing the questionnaire. All participants consented to eligibility and consented to publish the anonymized data before they could proceed to answer the questions, and contact details of the Charity and who was responsible for the survey and storage were provided. All responses were anonymous and no identifying personal information were requested. All responses were treated with the strictest confidentiality.

## Results

Responses from BSPED were received from 55 units from England and Wales, 2 from Scotland, and 2 from Northern Ireland. A total of 290 patients with recurrent hypoglycemia without diabetes sought to get access for CGM in the past 2 years with 226 from England and Wales, 12 from Northern Island, 28 from Wales, and 14 from Scotland. Tables 1 and 2 report on which specialties patients are referred to for CGM and how CGM funding was obtained.

**Figure 1.**
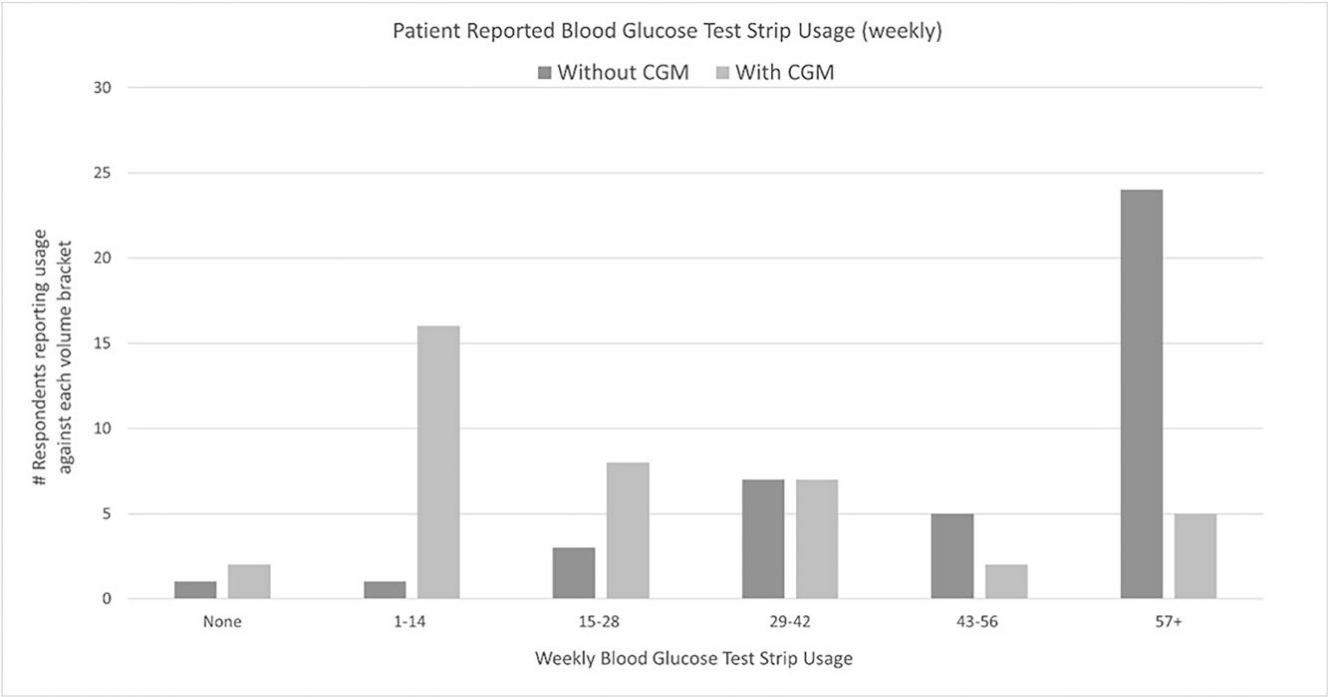
A comparison of patients with and without CGM reporting usage of weekly blood glucose test strips (Data derived from the UK CHC survey).

**Table 1.** In which specialty would a patient without diabetes be referred for a continuous glucose monitoring (CGM)? Data from BSPED survey

Location	Number of site responses	Consultant paediatric endocrinologist	Consultant paediatrician	Specialist nurse	Specialty doctor
England	45	14	25	5	1
Northern Ireland	3	1	1	1	0
Wales	5	4	0	1	0
Scotland	2	2	0	0	0
**Total**	**55**	**21**	**26**	**7**	**1**
**Location**	**General paediatric**	**Paediatric diabetes**	**Paediatric endocrinology**	**Paediatric metabolic**	**Other**
England	5	4	18	9	9
Northern Ireland	0	4	0	2	0
Wales	1	1	2	1	0
Scotland	0	0	2	0	0
**Total**	**6**	**9**	**22**	**12**	**9**

**Table 2.** How was funding obtained for the CGM in patients without diabetes? Data from BSPED survey

Location	Funded by charitable funds	Funded by trust	Funded directly by the CCG or local health board without a need for an IFR	Funding not sought	IFR to the CCGs or local health boards	Parent/carer self-funded	No information provided
England	1	5	2	8	22	0	4
Northern Ireland	0	2	0	1	0	1	1
Wales	0	2	0	3	1	1	1
Scotland	0	0	0	0	0	2	0
**Total**	**1**	**9**	**2**	**12**	**23**	**4**	**6**

Abbreviations: CCG, Clinical Commissioning Group; IFR, individual funding request.

The UK CHC survey received 62 responses from individual families (1 parent/carer responded per child), the largest response to a survey carried out by the charity. Table 3 shows the responses related to patients using finger-prick testing, CGM, and Libre Freestyle flash glucose monitoring systems and their funding routes. Only 19 out of the 62 respondents (31%) were able to access CGM routinely for recurrent hypoglycemia.

**Table 3.** UK CHC survey data looking at CGM access

Please indicate the type of blood glucose monitoring routinely used	Finger-prick testing	CGM	Libre Freestyle
England	35	16	3
Northern Ireland	1	2	0
Scotland	4	1	0
**Total**	**40**	**19**	**3**
**Have you any prior experience of CGM?**	**No- have only ever finger-pricked**	**Yes- have used a CGM**	
England	20	30	
Northern Ireland	1	2	
Scotland	3	2	
**Total**	**24**	**34**	

Quality of life benefits were evident from parents and carers on how CGM reduced worry, provided reassurance, improved sleep patterns, and improved quality of life such as ability to participate in school and social activities and leisure time. CGM systems were also noted to lessen the burden of frequently finger-pricking, reducing unplanned hospital admissions, and reducing out-of-hours appointments as a result of hypoglycemia (Tables 4 and 5).

**Table 4.** UK CHC survey in quality of life and benefits of CGM utilization

If you have used a CGM (current or previously), have you seen an improvement in the quality of life of the person who suffers from the episodes of hypoglycaemia	Survey option	Responses
	N/A I do not use a CGM	28
	Improved their quality of life	35
	No change in quality of life	4
	Worsening of their quality of life	1
	**Total**	**40**
If you have used a CGM (current or previously), have you seen an improvement in the quality of life of the parent/guardian/carer of the person who suffers from the episodes of hypoglycaemia?	Survey Option	Responses
	N/A I do not use a CGM	29
	Improved their quality of life	34
	No change in their quality of life	3
	Worsening of their quality of life	2
	**Total**	**39**
When using a CGM, have you reduced your usage of any of the following?	Survey Option	Responses
	Reduced unplanned urgent hospital visits	26
	Reduced urgent GP appointments due to hypoglycaemia	15
	Reduced out of hours appointments due to hypoglycaemia	16
	Reduced prescriptions eg, lancets, blood glucose test strips	25
Please mark from the options below all the parameters you believe you have seen an improvement on when using a CGM	Survey Option	Responses
	Less missed work or school days	29
	Able to manage blood glucose monitoring independently	28
	Ability to participate in social activities	36
	Ability to participate in sporting activities	29
	Improved sleep quality	35
	Reduction in anxiety ie, reduced fear of hypoglycaemia	39

**Table 5.** Parent/carer quotations (Data derived from the UK CHC survey)

Theme and sub-themes	Quotations
Improved quality of life benefits	“Been the best thing for my daughter, we all get sleep, and we always know when she need help with sugars its been a God send” “Peace of mind to be able to lead as “normal” a life as possible” “We can go on short walks now where before we could have none” “School can involve him more in sports with having the CGM. School can stretch him to a lunch break to eat with friends using the CGM to give boluses to safely get to lunch as opposed to a very isolated lunch break one hour earlier than everyone else. The benefits I could list forever!” “It is the best thing we have ever had to enable a better quality of life”
Clinical benefits of predicting and preventing hypoglycaemia occurrence	“We’ve had less ambulance call outs, less hospital stays and less episodes of severe hypoglycaemia. I can’t imagine life without it” “Great to give trend and prevent hypos” “Huge reduction in hospital visits/admissions, it also enables far better management and control of the condition on a daily basis and a vastly improved understanding of the blood sugar patterns”
Reassurance and less worry, improved sleep	“This is vital for those with hypoglycaemia. Makes things so much easier, lessens the worry of hypos- it should be available” “My daughter shows no outward signs of being hypoglycaemic and she has learning disabilities so is unable to tell us if she feels unwell. The CGM allows us and school to act to prevent her dropping too low. Without the CGM it was extremely difficult, and we would only discover hypos if they happened to be when we tested” “It takes away so much of the stress and worry, sleepless nights and gives us peace of mind. School and respite also feel far better prepared with the Dexcom.” “Using a CGM allows us to monitor our child remotely reducing anxiety of schooling and other activities.” “Enables us to sleep better without worrying a hypo would not be detected early enough.”
Reducing the need to always finger-prick	“It really helps us parents have and ease of mind and not having to finger prick our children as much” “Gives her independence, able to meet with friends and be happy without the constant worry to finger-prick and check levels”

Patient-reported utilization rates of blood glucose test strips per week were significantly reduced for those using CGM, with the most significant blood glucose test strips reductions reported in those patients using more than 57+ test strips (See Fig. 1).

## Discussion

CGM is currently limited and nationally funded in the NHS only within the scope of patients with a diagnosis of diabetes. In people with type 1 diabetes, there is strong evidence that CGM technology improves detection of asymptomatic hypoglycemia compared with intermittent blood finger-prick glucose monitoring, and that use of CGM prevents the frequency of hypoglycemic events [3]. However, there are currently no published trials on use of CGM in the population of children with recurrent hypoglycemia, such as in congenital hyperinsulinism or metabolic disorders. There is a likelihood that that trends on CGM would be very valuable to patients and their carers, but problems may still exist with CGM performance at very low glucose concentrations and these require further research. Research in patients with hyperinsulinism have shown detection of very low glucose concentrations and that early hours of the day is a time of highest hypoglycemic risk for people with hyperinsulinism [5]. In these circumstances, despite frequent intermittent blood glucose testing and the associated time burden, parents and carers will not be able to regularly detect hypoglycemia when their child is asleep. The UK CHC survey findings showed that the CGM had a significant positive impact in the daily lives of parents and carers. CGM technology was noted to offer a safety net for missed hypoglycemic episodes that were potentially life threatening, improved quality of life for the child and the family, and reduced their worries and anxieties. This was in line with clinical research in children with diabetes in which CGM devices were noted to alleviate worry and fear of hypoglycemia in children and caregivers [6]. The UK CHC survey also noted that less than a third of people have access to CGM technology, and many have stated that they simply cannot afford the ongoing cost to self-fund with rising cost of living.

CGM education is critical to support its use and barriers may include limited training in CGM use and interpretation of the CGM data. It is imperative that CGM initiation by healthcare professionals include education on how to use it effectively, and an understanding of its limitations when making clinical decisions based on the CGM metrics and downloads [7]. CGM technology has the potential to improve the quality of life for people with recurrent hypoglycemia, improve neurological outcomes, and reduce hospital admissions.

The prevention of recurrent hypoglycemia remains a cornerstone management for people who suffer from frequent hypoglycemia. The BSPED national survey reported that access to CGM funding for patients with recurrent hypoglycemia was highly variable between hospital providers and that funding of CGM lacks a standardized approach, with a low success rate of NHS funding. BSPED and UK CHC urge a review of CGM access to include people who suffer from recurrent hypoglycemia, without the diagnosis of diabetes. More research is warranted to determine the safety and efficacy of CGM in detection and reduction of hypoglycemic events, impact on hospital stay, and long-term neurological outcomes.

## Data Availability

This is available on request.

## Abbreviations

BSPED: British Society of Paediatric Endocrinology and Diabetes
CCG: Clinical Commissioning Group
CGM: continuous glucose monitoring
NHS: National Health Service
NICE: National Institute of Clinical Excellence
UK CHC: UK Children’s Hyperinsulinism Charity
